# CMR findings after COVID-19 and after COVID-19-vaccination—same but different?

**DOI:** 10.1007/s10554-022-02623-x

**Published:** 2022-05-12

**Authors:** Patrick Doeblin, Constantin Jahnke, Matthias Schneider, Sarah Al-Tabatabaee, Collin Goetze, Karl J. Weiss, Radu Tanacli, Alessandro Faragli, Undine Witt, Christian Stehning, Franziska Seidel, Ahmed Elsanhoury, Titus Kühne, Carsten Tschöpe, Burkert Pieske, Sebastian Kelle

**Affiliations:** 1 Department of Internal Medicine/Cardiology, Deutsches Herzzentrum Berlin, Berlin, Germany; 2 DZHK (German Centre for Cardiovascular Research), Partner Site Berlin, Berlin, Germany; 3 Department of Cardiology, Campus Virchow Klinikum, Charité – Universitätsmedizin Berlin, Corporate Member of Freie Universität Berlin and Humboldt-Universität zu Berlin, Charitéplatz 1, 10117 Berlin, Germany; 4 Clinical Science, Philips Healthcare, Hamburg, Germany; 5 Department of Congenital Heart Disease, German Heart Center Berlin, Berlin, Germany; 6 BIH Berlin Institute of Health at Charite (BIH), BIH Center for Regenerative Therapies (BCRT) Universitätsmedizin Berlin, Berlin, Germany

**Keywords:** CMR, COVID-19, Long COVID, COVID-19 vaccines, Myocarditis, Pericarditis

## Abstract

Cardiac involvement has been described in varying proportions of patients recovered from COVID-19 and proposed as a potential cause of prolonged symptoms, often described as post-COVID or long COVID syndrome. Recently, cardiac complications have been reported from COVID-19 vaccines as well. We aimed to compare CMR-findings in patients with clinical cardiac symptoms after COVID-19 and after vaccination. From May 2020 to May 2021, we included 104 patients with suspected cardiac involvement after COVID-19 who received a clinically indicated cardiac magnetic resonance (CMR) examination at a high-volume center. The mean time from first positive PCR to CMR was 112  ± 76 days. During their COVID-19 disease, 21% of patients required hospitalization, 17% supplemental oxygen and 7% mechanical ventilation. In 34 (32.7%) of patients, CMR provided a clinically relevant diagnosis: Isolated pericarditis in 10 (9.6%), %), acute myocarditis (both LLC) in 7 (6.7%), possible myocarditis (one LLC) in 5 (4.8%), ischemia in 4 (3.8%), recent infarction in 2 (1.9%), old infarction in 4 (3.8%), dilated cardiomyopathy in 3 (2.9%), hypertrophic cardiomyopathy in 2 (1.9%), aortic stenosis, pleural tumor and mitral valve prolapse each in 1 (1.0%). Between May 2021 and August 2021, we examined an additional 27 patients with suspected cardiac disease after COVID-19 vaccination. Of these, CMR provided at least one diagnosis in 22 (81.5%): Isolated pericarditis in 4 (14.8%), acute myocarditis in 9 (33.3%), possible myocarditis (acute or subsided) in 6 (22.2%), ischemia in 3 (37.5% out of 8 patients with stress test), isolated pericardial effusion (> 10 mm) and non-compaction-cardiomyopathy each in 1 (3.7%). The number of myocarditis diagnoses after COVID-19 was highly dependent on the stringency of the myocarditis criteria applied. When including only cases of matching edema and LGE and excluding findings in the right ventricular insertion site, the number of cases dropped from 7 to 2 while the number of cases after COVID-19 vaccination remained unchanged at 9. While myocarditis is an overall rare side effect after COVID-19 vaccination, it is currently the leading cause of myocarditis in our institution due to the large number of vaccinations applied over the last months. Contrary to myocarditis after vaccination, LGE and edema in myocarditis after COVID-19 often did not match or were confined to the RV-insertion site. Whether these cases truly represent myocarditis or a different pathological entity is to be determined in further studies.

## Background

### Cardiac complications during the acute phase of COVID-19

The SARS-CoV-2 epidemic has strained the resources of health care systems and judicious use of advanced imaging modalities is necessary to prevent overburdening. While the primary target of SARS-CoV-2 is the respiratory system, cardiac complications have been reported in varying frequency [1]. An early observational study from Wuhan reported troponin I elevations in 37.5% and clinically suspected myocarditis in 12.5% of hospitalized COVID-19 patients based on symptoms, electrocardiography (ECG) and echocardiography [2]. Subsequent histopathological studies found any cardiac pathology in 47.8% of deceased patients but myocarditis in less than 2% [3]. Other reported findings were pericarditis, macro- and microthrombi, “nonmyocarditis” inflammation and acute myocardial infarction [3, 4].

### Persistent cardiopulmonary symptoms after COVID-19

Persistent cardiopulmonary symptoms after the acute phase of COVID-19 are common. In a study of 143 hospitalized COVID-19 patients in Italy, 86.4% were still symptomatic at 2 months after symptom onset [5]. Common complaints were fatigue, dyspnea and chest pain. Another study of 1733 hospitalized patients in Wuhan in early 2020 with a median follow up of 6 months showed persistent fatigue in 63%, palpitations in 9% and chest pain in 5% of patients [6]. A prospective study of 247 Norwegian home-isolated patients found persistent fatigue (30%), dyspnea (15%) and palpitations (6%) at 6 months follow up [7]. The British National Institute for Health and Care Excellence guideline on the managing of the long-term effects of COVID-19 recommends the term “ongoing symptomatic COVID-19” for symptoms 4 to 12 weeks and “post-COVID-19 syndrome” for symptoms more than 12 weeks after the start of acute COVID-19 [8]. Another commonly used term is “long COVID” [7].

### Cardiac magnetic resonance (CMR) in COVID-19

CMR offers the non-invasive assessment of several cardiac pathologies including myocarditis, pericarditis and embolic complications. CMR studies in patients recovered from the acute phase of COVID-19 have reported widely differing proportions of myocarditis after COVID-19 ranging from 0 to 60% of patients owing mostly to different diagnostic criteria and patient selection [1]. The International Consensus Group on CMR Diagnosis of Myocarditis published a white paper in 2009, proposing three “Lake Louise Criteria” (LCC) consisting of edema, early enhancement and LGE, two of which were required for a CMR-diagnosis of myocarditis [9]. A 2018 update of those criteria dropped early enhancement and incorporated T1-, ECV and T2-mapping-techniques as alternative parameters of fibrosis and edema, with one fibrosis- and one edema-criterion required for a CMR-diagnosis of myocarditis [10]. Most studies published so far have not consistently applied these criteria, rendering comparisons challenging [11–25]. Moreover, most published studies have examined competitive athletes, which are not representative of the patient collective in daily clinical practice [11, 13–15, 19, 20, 22–24]. To date there are only small case series on the prevalence of myocarditis in non-athletes which consistently applied the updated Lake-Louise-criteria. Weckbach et al. examined 18 patients with elevated troponin, seven of which had myocarditis [25]. Huang et al. examined 26 patients with cardiac symptoms, seven of which had myocarditis [16]. A study by Chen et al. in 25 acute hospitalized COVID-19 patients found myocardial edema in 14 and myocardial damage in one patient [12]. A study in 18 asymptomatic pediatric patients after mild COVID-19 found no apparent cardiac involvement by CMR [26].

### Cardiac complications after COVID-19-vaccination

Since early 2021, reports of myocarditis after COVID-19-Vaccination have surfaced, mostly considered ‘mild’ with rapid clinical improvement [27–34]. Unless stated otherwise, ´vaccination´ in this paper denominates COVID-19-vaccination. Case-series reported resolution of edema and reduction of the size of LGE on follow-up CMR [35, 36]. While the pathophysiologic mechanism remains unclear and the number of cases is low compared to the number of vaccinations, the close temporal correlation with higher than expected absolute case numbers suggests a causal connection [37, 38].

### Aim of the current study

We aimed to examine the prevalence of cardiac findings on CMR in a representative clinical collective of patients referred for suspected cardiac involvement after COVID-19 and vaccination.

## Methods

We searched our hospital database for patients that underwent a clinical CMR examination between May 2020 and May 2021 for suspected cardiac pathology after COVID-19. For comparison, CMR examinations from January until August 2021 were searched for suspected cardiac pathology after COVID-19 vaccination. In case of multiple COVID-19 vaccinations, the last vaccination preceding symptoms was suspected as the causative vaccination. The study complies with the declaration of Helsinki and was approved by the ethics committee of the Charité-Universitätsmedizin Berlin (EA2/020/21) with a waiver of consent. It is registered at ClinicalTrials.gov (NCT05124223). A subset of the patients was published earlier as case series [34, 39].

### CMR imaging

All scans were performed for clinical indications on either a Philips Ingenia 3.0T scanner or a Philips Ambition 1.5T scanner (Koninklike Philips N.V., Amsterdam, The Netherlands) according to recent recommendations [1, 10, 40]. Protocols were adjusted to the clinical scenario but generally included standard CINE imaging, T2 STIR edema imaging, T2 mapping (T2-GraSE), pre- and post-contrast T1 mapping (MOLLI), and Late-Enhancement-Imaging (mDIXON) [39]. Vasodilator stress with Regadenosone or Adenosine was performed as clinically indicated in patients with suspected myocardial ischemia. The contrast agent dose was 0.1–0.15 mmol/kg Gadobutrol (Gadovist®, Bayer AG, Leverkusen, Germany) [41].

### CMR image analysis

Image post-processing and measurements were performed according to recent recommendations using dedicated CMR post-processing software (IntelliSpace Portal V11.1, Koninklike Philips N.V., Amsterdam, The Netherlands) [42]. The diagnosis of acute myocarditis was based on the updated Lake Louise Criteria (LLC) requiring findings of myocardial damage (non-ischemic LGE) and edema (T2 STIR or T2 mapping) in a non-ischemic pattern (intramyocardial or subepicardial). Patients fulfilling only one LLC criterion were considered possible myocarditis, further divided into edema without myocardial damage as ‘possible acute myocarditis’ and myocardial damage without edema as ‘possible subsided myocarditis’.

### Statistical analysis

Statistical analysis was performed using SPSS 25 (IBM, Armonk, NY, USA) and RStudio (RStudio PBC, Boston, MA, USA). Baseline data were reported as means ± standard deviations (SD) for interval- and ratio-scaled parameters and as numbers and percentages for nominal and ordinal-scaled parameters.

## Results

### Baseline characteristics

The baseline characteristics of post-COVID-19 and post-vaccination patients are given in Table 1. About four times more patients were referred for suspected cardiac involvement after COVID-19 than after vaccination. Post-COVID-19 patients were slightly older (47.6 ± 14.0 vs. 43.9 ± 20.4 years) and had more comorbidities than post-vaccination patients. Post-vaccination patients more often complained of cardiac symptoms at the time of CMR compared to post-COVID-19 patients (92.6% vs. 55.8%) and more frequently presented with elevated troponin (42.1 vs. 27.0%), although data on the latter was missing in many post-COVID-patients. The time from suspected causative event to CMR was longer in the post-COVID-19 patients (time from first positive PCR to CMR, 112 ± 76 days) than in post-vaccination patients (time from vaccine to CMR, 44 ± 35 days).

**Table 1:** Baseline characteristics of post-COVID-19- and post-vaccine-patients

Parameter	Unit	Post COVID-19 N = 104		Post vaccine N = 27	
*Biometric data*					
Sex	Female	41	(39.4%)	8	(29.6%)
Age	Years	47.6	± 14.0	43.9	± 20.4
BMI	kg/m^2^	26.3	± 6.2	23.7	± 3.2
*Medical history*					
Arterial hypertension		28	(26.9%)	6	(22.2%)
Diabetes mellitus		6	(5.8%)	1	(3.7%)
Dyslipidemia		15	(14.4%)	1	(3.7%)
Coronary artery disease		14	(13.5%)	2	(7.4%)
Smoking (current or former)		40	(38.5%)		N/A
Asthma/COPD		11	(10.6%)	2	(7.4%)
N/A		2	(1.9%)	1	(3.7%)
*Medication*					
Aspirin		15	(14.4%)	1	(3.7%)
Oral anticoagulant		11	(10.6%)	2	(7.4%)
Statin		11	(10.6%)	3	(11.1%)
Beta-blocker		25	(24.0%)	6	(22.2%)
Diuretics		17	(16.3%)	3	(11.1%)
ACE-inhibitor		19	(18.3%)	1	(3.7%)
AT2-antagonist		16	(15.4%)	3	(11.1%)
Calcium-antagonist		7	(6.7%)	2	(7.4%)
N/A		5	(4.8%)	1	(3.7%)
*Severity of COVID-19*					
Ambulatory		79	(76.0%)		
Hospitalized		21	(20.2%)		
Supplementary oxygen		17	(16.3%)		
Mechanical ventilation		7	(6.7%)		
N/A		4	(3.8%)		
*Laboratory values (median and range)*					
hsCRP	mg/dL	0.25	0.0–34.2 (N = 39)	0.345	0.1–68.6 (N = 12)
hsCRP elevated	> 0.5 mg/dL	9	(23.1%, N = 39)	5	(41.7%, N = 12)
hsTnI	pg/mL	1.1	0.0–67.0 (N = 18)	4834	0.3–14,934.7 (N = 6)
hsTnT	ng/L	8.5	2–189 (N = 18)	7.3	3–2000 (N = 9)
hsTn elevated (I or T)^a^		10	(27.0%, N = 37)	8	(42.1%, N = 19)
NT-proBNP	pg/mL	53.9	10–9606 (N = 40)	351	8–807 (N = 7)
BNP/NT-proBNP elevated^b^		10	(25.0%, N = 40)	4	(40.0%, N = 10)
*Cardiac symptoms at time of CMR*					
Chest pain		36	(34.6%)	21	(77.8%)
Dyspnea		41	(39.4%)	16	(59.3%)
Palpitations		12	(11.5%)	6	(22.2%)
Any of the above		58	(55.8%)	26	(96.3%)
N/A		2	(1.9%)	1	(3.7%)
Time from first positive PCR to CMR	Days	112	± 76		
Time from vaccine to CMR	Days			44	± 35
Time from vaccine to symptoms	Days			13	± 12 (N = 26)
Time from symptoms to CMR	Days			35	± 35 (N = 26)
*Suspected causative vaccine*					
Pfizer/BioNTech				19	(70.4%)
Moderna				7	(25.9%)
AstraZeneca				1	(3.7%)

*ACE* angiotensin converting enzyme, *AT2* angiotensin 2, *BMI* body mass index, *CMR* cardiac magnetic resonance, *COPD* chronic obstructive pulmonary disease, *N/A* not available, *PCR* polymerase chain reaction

^a^Upper limit of normal (99% percentile) for hsTnT 14 ng/L, for hsTnI 17 pg/ml for females and 35 pg/ml for males. For four patients, only qualitative information on troponin elevation was available

^b^Upper limit of normal for NT-proBNP dependent on sex and age

### CMR results

CMR results are presented in Table 2 and the final CMR diagnoses in Table 3. The majority of post-COVID-19 patients were scanned on a 3 Tesla scanner whereas post-vaccination patients were scanned equally on 1.5 and 3 Tesla scanners. While the number of patients with suspected cardiac involvement after vaccination was smaller than after COVID-19, the prevalence of findings in patients after vaccination was higher. In 34 (32.7%) of post-COVID-19 patients, CMR provided a clinically relevant diagnosis: Isolated pericarditis in 10 (9.6%), acute myocarditis (both LLC) in 7 (6.7%), possible myocarditis (one LLC) in 5 (4.8%), ischemia in 4 (3.8%), recent infarction in 2 (1.9%), old infarction in 4 (3.8%), dilated cardiomyopathy in 3 (2.9%), hypertrophic cardiomyopathy in 2 (1.9%), aortic stenosis, pleural tumor and mitral valve prolapse each in 1 (1.0%). In post-vaccination patients, CMR provided at least one diagnosis in 22 (81.5%): Isolated pericarditis in 4 (14.8%), acute myocarditis in 9 (33.3%), possible myocarditis (acute or subsided) in 6 (22.2%), ischemia in 3 (37.5% out of 8 patients with stress test), isolated pericardial effusion (> 10 mm) and non-compaction-cardiomyopathy each in 1 (3.7%).

**Table 2:** CMR results

Parameter	Unit	Post-COVID (N = 104)	Post-vaccination (N = 27)	Normal	*P* value
1.5 Tesla		7 (6.6%)	14 (51.9%)		
3 Tesla		97 (93.4%)	13 (48.1%)		
LVEF	%	60.5 ± 8.9	58.9 ± 6.8	57–77^a^	0.395
LVEDVi	mL/m^2^	76.7 ± 16.8	78.5 ± 15.9	57–105^a^	0.616
RVEF	%	52.6 ± 6.7 (N = 99)	56.8 ± 5.6 (N = 19)	52–72^a^	0.010
RVEDVi	mL/m^2^	79.1 ± 16 (N = 99)	83.6 ± 17.3 (N = 19)	61–121^a^	0.251
LA	cm^2^	20.5 ± 5.2	20.2 ± 4.4	15–29^a^	0.766
RA	cm^2^	22.6 ± 5.2	21.3 ± 4.9	14–30^a^	0.248
T1 (3T)	ms	1229 ± 65 (N = 97)	1241 ± 32 (N = 13)	1173–1334^b^	0.487
T1 (1.5T)	ms	983 ± 24 (N = 7)	1038 ± 87 (N = 14)	903–1085^b^	0.119
T2 (3T)	ms	46.3 ± 5.2 (N = 94)	43.5 ± 1.8 (N = 11)	35–51^b^	0.087
T2 (1.5T)	ms	50.7 ± 2.3 (N = 6)	50.6 ± 3.9 (N = 14)	41–57^b^	0.989
ECV	%	24.9 ± 3.1 (N = 95)	25.9 ± 3.8 (N = 24)	≤ 30	0.198
LGE ischemic		8 (7.7%)	0 (0%)		0.137
LGE non-ischemic		14 (13.5%)	17 (63.0%%)		< 0.001
N° of segments		1.6 ± 1.2 (N = 14)	2.4 ± 1.5 (N = 17)		0.134
Regional edema		7 (4.8%)	9 (33.3%)		< 0.001
Global edema		4 (3.8%)	1 (3.7%)		0.973
Pericardial LGE/edema		13 (12.5%)	5 (17.9%)		0.418
LVEF < 50%		14 (10.6%)	2 (7.4%)		0.160
Regional WMA		12 (11.5%)	5 (18.5%)		0.356
RVEF < 45%		9 (8.7%)	0 (0%)		0.289
Pericardial effusion (> 3 mm)		6 (5.8%)	3 (11.1%)		0.328

*CMR* cardiac magnetic resonance, *ECV* extracellular volume, *LA* left atrium, *LGE* late gadolinium enhancement, *LVEDVi* left ventricular enddiastolic volume index, *LVEF* left ventricular ejection fractionRA right atrium, *RVEDVi* right ventricular enddiastolic volume index, *RVEF* right ventricular ejection fraction, *T1* basomedial septal T1 relaxation time, *T2* basomedial septal T2 relaxation time, *WMA* wall motion abnormalities

^a^Normal values from Kawel-Boehm et al. (2020)

^b^Local normal values from unpublished data

**Table 3:** CMR diagnosis of patients referred for suspected cardiac involvement

Diagnosis	Post-COVID (N = 104)	Post-vaccination (N = 27)
Acute myocarditis (Myocardial edema with non-ischemic LGE)	7 (6.7%)	9 (33.3%)
Perimyocarditis	1 (1.0%)	1 (3.7%)
Isolated myocarditis	6 (5.8%)	8 (29.6%)
Possible acute myocarditis (Myocardial edema without non-ischemic LGE)	2 (1.9%)	0 (0%)
Perimyocarditis	1 (1.0%)	0 (0%)
Isolated myocarditis	1 (1.0%)	0 (0%)
*Possible subsided myocarditis*		
Non-ischemic LGE without myocardial edema and no other apparent cause	3 (2.9%)	6 (22.2%)
Isolated pericarditis	10 (8.7%)	4 (14.8%)
Ischemia (stress-induced perfusion deficit)	4 (6.3%, N = 63)	3 (37.5%, N = 8)
Recent infarction (ischemic LGE with edema)	2 (1.9%)	0 (0%)
Old infarction (ischemic LGE without edema)	4 (3.8%)	0 (0%)
Micro-infarction (old or recent)	3 (2.9%)	0 (0%)
DCM	3 (2.9%)	0 (0%)
HCM	2 (1.9%)	0 (0%)
Severe aortic stenosis	1 (1.0%)	0 (0%)
Pleural tumor	1 (1.0%)	0 (0%)
Mitral valve prolapse	1 (1.0%)	0 (0%)
Isolated pericardial effusion > 10 mm	0 (0%)	1 (3.7%)
Non-compaction cardiomyopathy	0 (0%)	1 (3.7%)
Any diagnosis	34 (32.7%)	22 (81.5%)

*CMR* cardiac magnetic resonance, *DCM* dilated cardiomyopathy, *HCM* hypertrophic cardiomyopathy, *LGE* late gadolinium enhancement

**Fig. 1:**
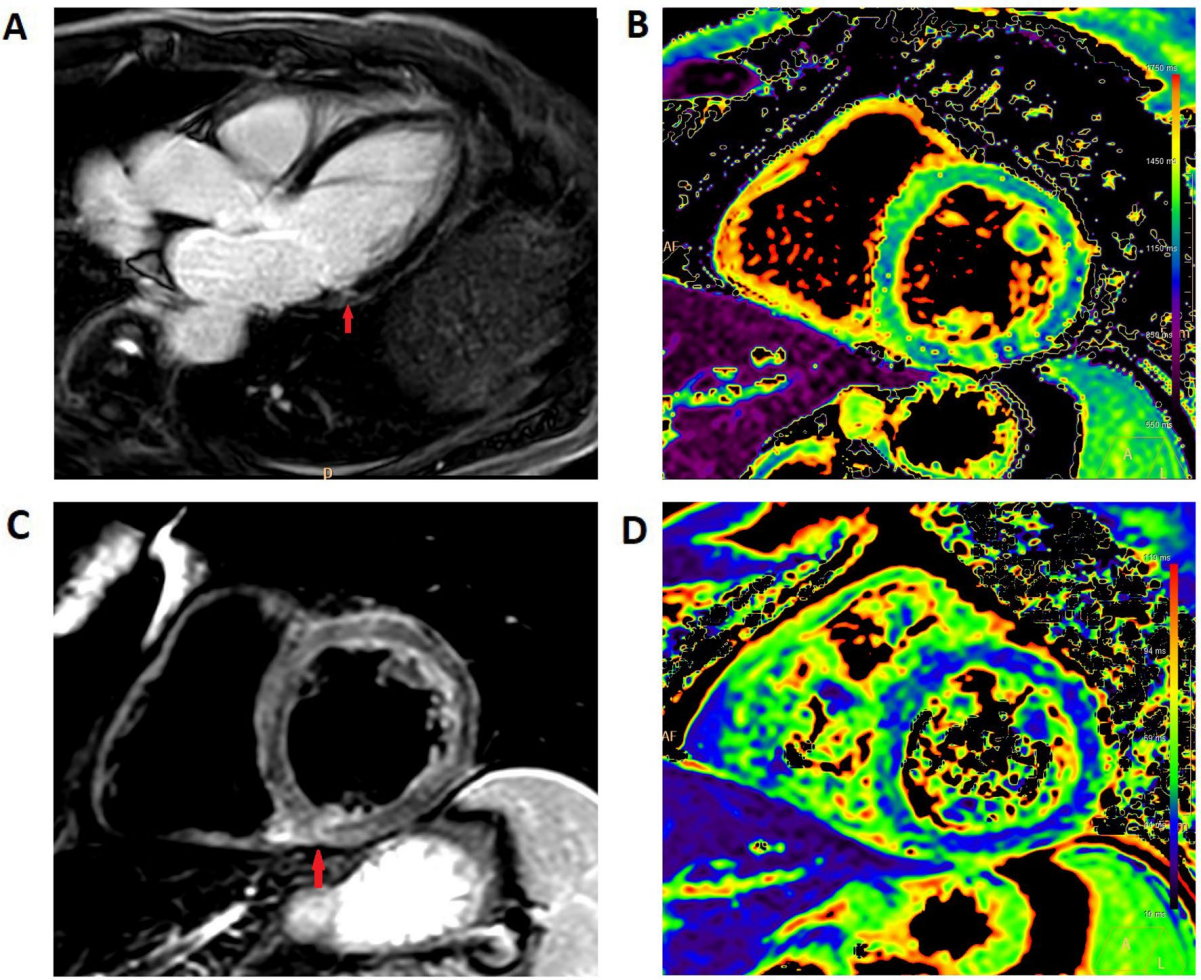
CMR images of a patient with myocarditis post-COVID (patient C5). **A** 3 chamber view, late gadolinium enhancement shows a subepicardial fibrosis inferolateral basal (arrow). **B** Medial short axis, T1 mapping shows no evidence of diffuse fibrosis. **C** Basal short axis, T2 STIR imaging showed edema at the inferior RV-insertion-site (arrow). **D** Medial short axis, T2 mapping shows no evidence of global edema

**Fig. 2:**
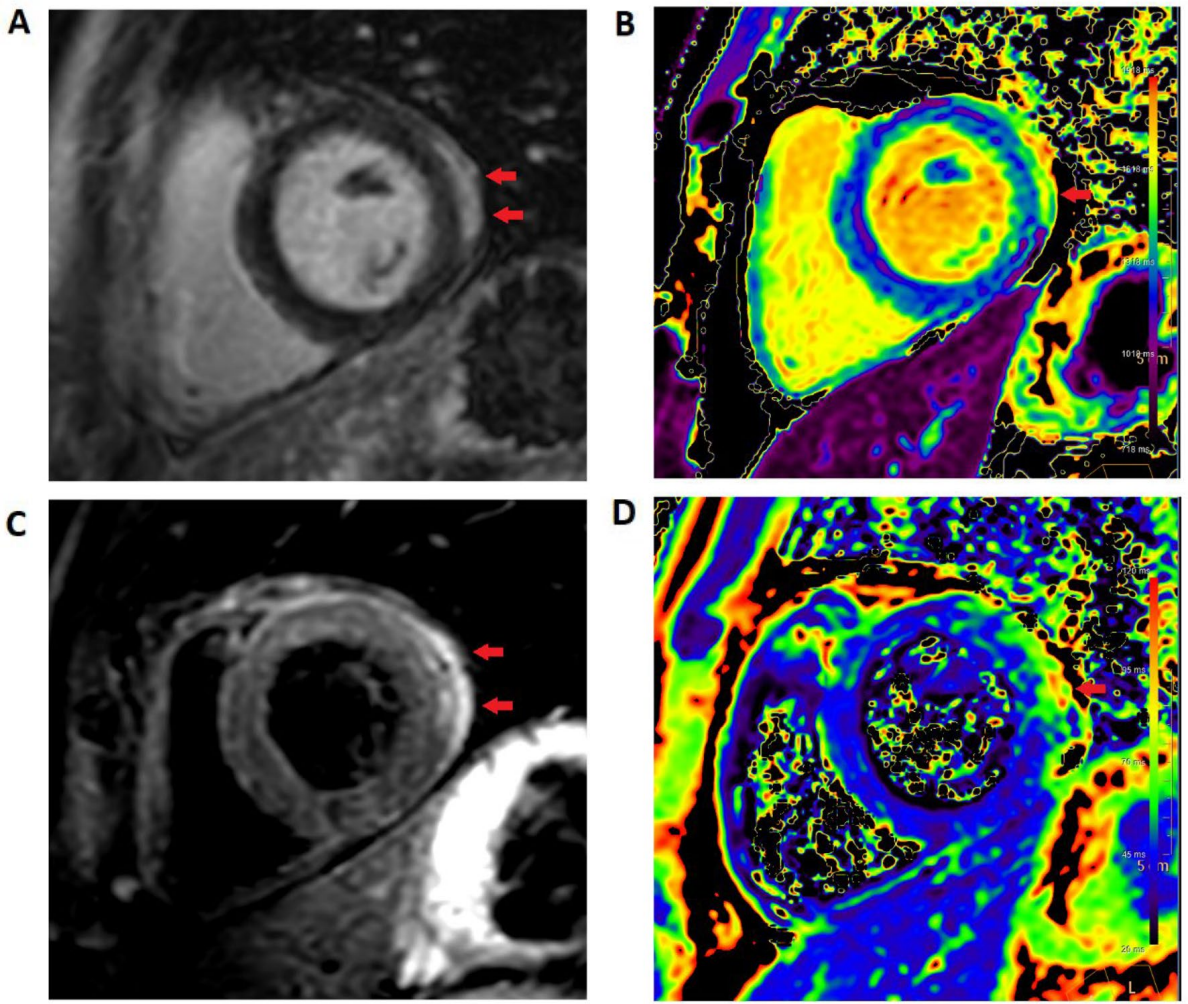
CMR images of a patient with perimyocarditis post COVID-Vaccine (Patient V1). All images medial short axis. **A** Late gadolinium enhancement shows subepicardial fibrosis with pericardial involvement. **B** T1 mapping shows focal but not diffuse fibrosis. **C** T2 STIR imaging shows subepicardial and pericardial edema (arrows). **D** T2 mapping shows no evidence of global edema

The clinical characteristics of all patients with acute myocarditis or possible acute myocarditis are listed in Table 4 for post-COVID-19 and in Table 5 for post-vaccination patients. In all 13 Patients with acute myocarditis on CMR and data on ECG, echocardiography and troponin, at least one of the latter was abnormal. Figures 1 and 2 show exemplary CMR images of post-COVID-19 patients and post-vaccination patients with probable acute myocarditis.

**Table 4:** Clinical characteristics of patients with acute myocarditis and possible acute myocarditis after COVID-19 on CMR

Pt	Age	Sex	Symptoms	Echo	ECG	Lab	CMR findings	CMR diagnosis	Biopsy	CMR follow up
C1	60+	m	Dyspnea	WMA	AFlu	NT-proBNP	Intramural LGE inferolateral basal, globally elevated T2, RVEF 41%	Acute myocarditis	N/A	Resolved
C2	20+	m	Dyspnoe	–	ST	–	Globally elevated T2, LVEF 42%, RVEF 33%	Possible acute myocarditis	Subsiding Myocarditis	Resolved
C3	20+	w	Chest pain	N/A	N/A	N/A	Regionally elevated T2, focal pericarditis	Possible acute myocarditis, pericarditis	N/A	N/A
C4	60+	m	–	WMA	–	NT-proBNP	Intramural LGE inferoseptal basal, globally elevated T1, T2 and ECV, LVEF 39%, RVEF 45%	Acute myocarditis	No Myocarditis	Resolved
C5	50+	m	Chest pain	WMA	–	–	Subepicardial LGE and Edema inferolateral basal, LVEF 51%	Acute myocarditis	Slight interstitial fibrosis	Resolved
C6	30+	m	Chest pain	WMA	–	–	Intramural LGE inferior RV insertion site with matching edema, focal pericarditis	Acute myocarditis, pericarditis	N/A	Pericarditis resolved
C7^a^	40+	m	Palpitations	WMA	LBBB	N/A	Subepicardial LGE and Edema inferoseptal medial, LVEF 40%, small ischemia anterior apical	Acute myocarditis	N/A	Persistent LBBB
C8	40+	w	Dyspnea	N/A	N/A	N/A	Intramural LGE inferior RV insertion site with matching edema, mitral valve prolapse	Acute myocarditis	N/A	N/A
C9	70+	w	Chest pain	WMA	STD	Trop	Acute myocardial infarction inferolateral basal, LGE and edema inferior RV insertion site, subepicardial LGE inferolateral medial and inferior apical	Acute myocardial infarction, Acute myocarditis	N/A	N/A

– no abnormality detected, *AFlu* atrial flutter, *ECV* extracellular volume, *LBBB* left bundle branch block, *LGE* late gadolinum enhancement, *LVEF* left ventricular ejection fraction, *N/A* not available, *RV* right ventricle, *RVEF* right ventricular ejection fraction, *ST* sinus tachycardia, *STD ST *depression, *WMA* wall motion abnormalities (regional or global)

^a^Patient C7 had intermittent LBBB before and persistent LBBB with CMR evidence of myocarditis after COVID-19. All other findings besides the LBBB resolved on follow up

**Table 5:** Clinical characteristics of patients with possible or probable myocarditis after COVID-19 vaccination on CMR

Pt	Age	Sex	Symptoms	Echo	ECG	Lab	CMR findings	CMR diagnosis	Biopsy	Vaccine
V1	20+	m	N/A	–	–	Trop	Subepicardial LGE and edema basal and medial lateral wall	Acute myocarditis	Subsided myocarditis	2. Moderna (1. AZ)
V2	50+	m	Chest pain	–	STE	Trop	Subepicardial LGE and edema basal and medial lateral and inferor wall	Acute myocarditis	N/A	2. Moderna (1. Moderna)
V3	40+	m	Chest pain, dyspnea	–	–	Trop	Subepicardial LGE and edema basal inferior and lateral wall	Acute myocarditis	N/A	2. BioNTech (1. AZ)
V4	20+	w	Dyspnea	PE	ST	Trop	Edema lateral and inferior wall, PE 14 mm, globally elevated T1, T2 and ECV,	Acute myocarditis	N/A	2. Moderna (1. Moderna)
V5	50+	w	Chest pain	PE	N/A	–	Transmural LGE and edema basal inferior and lateral wall	Acute myocarditis	N/A	2. BioNTech (1. AZ)
V6	20+	m	Chest pain	–	ST	Trop	Subepicardial LGE and edema basal inferior and lateral wall	Acute myocarditis	N/A	2. BioNTech (1. Biontech)
V7	20+	m	Chest pain	–	–	Trop	Subepicardial LGE and edema basal inferior and lateral wall	Acute myocarditis	N/A	2. Moderna (1. Moderna)
V8	30+	w	Chest pain, dyspnea	N/A	N/A	–	Subepicardial LGE and edema basal inferior and lateral wall	Acute myocarditis	N/A	1. BioNTech
V9	70+	m	N/A	N/A	N/A	N/A	Subepicardial LGE and edema basal lateral wall	Acute myocarditis	N/A	N/A

– no abnormality detected, *AFlu* atrial flutter, *AZ* AstraZeneca, *ECV* extracellular volume, *LBBB* left bundle branch block, *LGE* late gadolinum enhancement, *LVEF* left ventricular ejection fraction, *N/A* not available, *PE* pericardial effusion, *Pt* patient, *RV* right ventricle, *RVEF* right ventricular ejection fraction, *ST* sinus tachycardia, *STD ST *depression, *WMA* wall motion abnormalities

### Time course of myocarditis cases

**Fig. 3:**
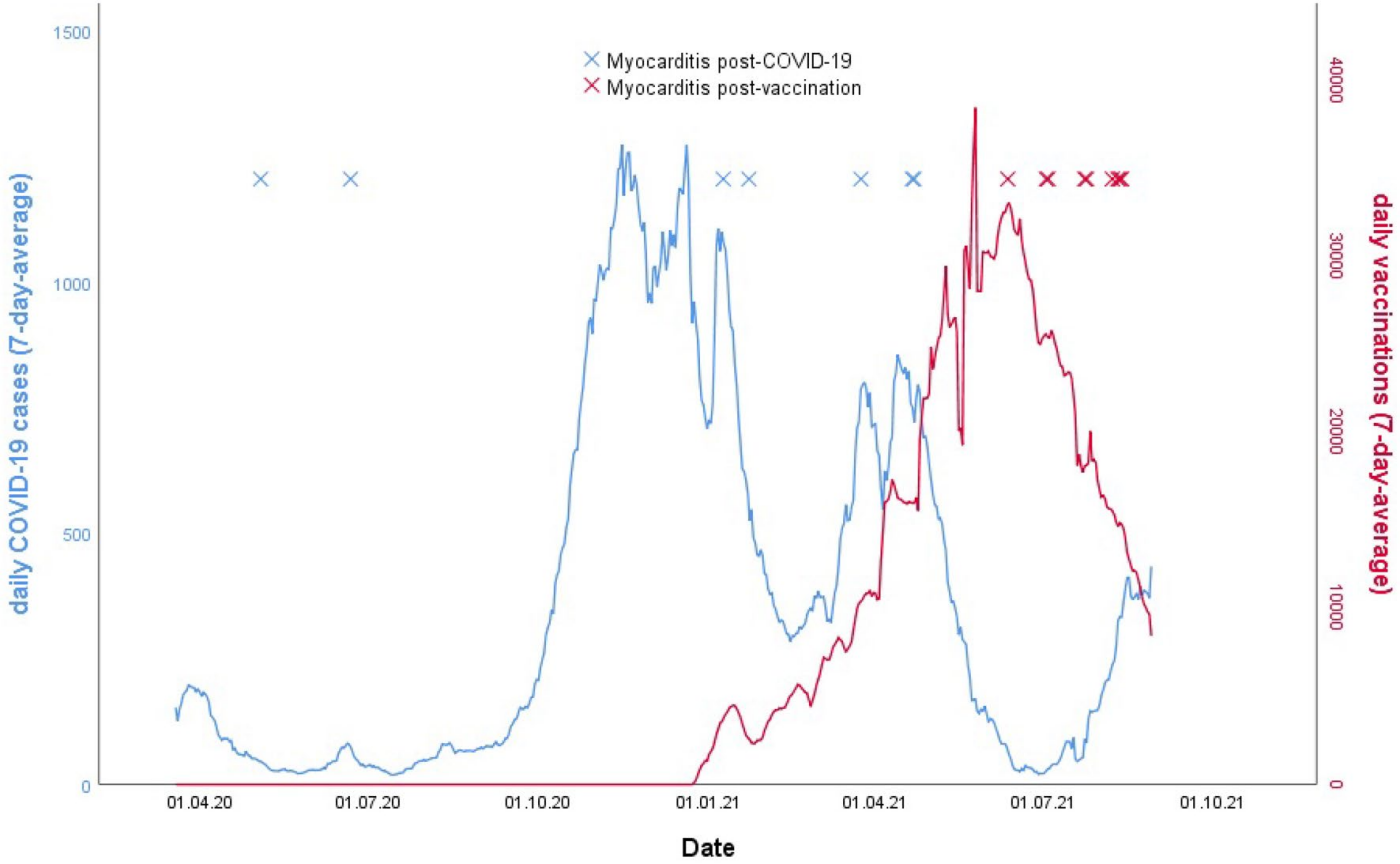
Daily cases of COVID-19 (blue, left axis) and vaccinations (red, right axis) in Berlin from April 2020 to August 2021. Overlaid on top the cases of Myocarditis after COVID-19 (blue crosses) and after vaccination (red crosses)

Quarterly cases of probable acute myocarditis and subsided myocarditis post COVID-19 and post vaccination are shown in Fig. 3a. For context, the monthly numbers of COVID-19-cases and vaccinations are shown in Fig. 3b [43, 44].

## Discussion

### Summary of findings

We analyzed 104 patients after COVID-19 and 27 patients after COVID-19-vaccination referred for CMR due to suspected cardiac involvement. The most frequent findings were

- Post COVID-19: 10 (8.7%) isolated pericarditis, 7 (6.7%) acute myocarditis (both LLC), 5 (4.8%) possible myocarditis (one LLC), 4 Ischemia (6.3% out of 63 with stress test) and 4 (3.8%) old myocardial infarction (all in patients with known coronary artery disease).
- Post vaccination: 9 (33.3%) acute myocarditis (both LLC), 6 (22.2%) possible myocarditis (one LCC), 4 (14.8%) isolated pericarditis and 3 (37.5% of 8 with stress test) ischemia.

These numbers are to be interpreted against the backdrop of 178,223 COVID-19 cases (per May 31th 2021) and 4,480,385 vaccination doses administered (per August 31th 2021) in Berlin (Fig. 3).

### Interpretation

COVID-19 causes cardiac symptoms in a substantial number of patients, explaining the higher number of referred patients post-COVID-19 compared to post-vaccination. Unfortunately, this referral did not lead to a specific diagnosis in the majority of cases after COVID-19, underlining our incomplete understanding of long COVID. The absolute number of myocarditis and pericarditis diagnoses was similar after COVID-19 and after vaccination, despite the far lower number of COVID-19 cases than vaccinations in Berlin. For perspective, the total number of myocarditis cases examined by CMR at our institution in 2019 was 17. Compared to myocarditis cases after vaccination, myocarditis cases after COVID-19 showed a less typical appearance. Localized, matching edema and LGE excluding the inferoseptal insertion point was present in only two of the seven cases after COVID-19 compared to nine of nine cases after vaccination. All cases of CMR diagnosed acute myocarditis had abnormalities on either ECG, echocardiography or troponin, when those were available.

### Comparison with other studies

Our findings are in line with those of other groups using the same diagnostic criteria and patients with clinical indication for CMR post-COVID-19 (Table 6) [11, 13–25]. The largest study to date, a retrospective analysis of 1597 screening-CMRs performed in athletes, reported CMR findings consistent with myocarditis in 37 (2.3%) participants. Seventeen (1.1% of total) of these had clinical suspicion of myocarditis based on symptoms or cardiac screening tests and 12 (0.8% of total) of those fulfilled both updated Lake-Louise-criteria [14].

**Table 6:** Studies on the prevalence of myocardial and pericardial involvement after COVID-19, sorted by patient population and indication for CMR

First author	Epub date	N CMR	LLC	MC (% of CMR)	PC (% of CMR)
*Non-athletes, screening*					
Puntmann	07 2020	100	−	60 (60%)	22 (22.0%)
*Non-athletes, suspected myocarditis*					
Knight	07 2020	51	−	13 (25.5%)	N/A
Huang	08 2020	26	+	7 (26.9%)	N/A
Kotecha	02 2021	148	−	40 (27.0%)	N/A
Weckbach	02 2021	18	+	7 (38.9%)	N/A
*Athletes, screening*					
Rajpal	09 2020	26	+	4 (15.4%)	N/A
Clarc	12 2020	59	+	2 (3.4%)	1 (1.7%)
Vago	12 2020	12	+	0 (0.0%)	0 (0.0%)
Starekova	01 2021	145	+	2 (1.4%)	1 (0.7%)
Moulson	04 2021	198	(+)	2 (1.0%)	2 (1.0%)
Daniels	05 2021	1597	(+)	37 (2.3%)	N/A
*Athletes, suspected myocarditis*					
Brito	11 2020	48	+	0 (0.0%)	19 (39.6%)
Martinez	03 2021	30	−	3 (10.0%)	2 (6.7%)
Moulson	04 2021	119	(+)	9 (7.5%)	6 (5.0%)
Hendrickson	05 2021	5	+	0 (0.0%)	0 (0.0%)

+ fulfilled, (+) mostly fulfilled, − not fulfilled, *LLC* Lake-Louise criteria, *MC* myocarditis, *N/A* not available, *PC* pericarditis. Cases of myopericarditis were counted as both MC and PC

The frequency of pericarditis was inconsistently reported in published CMR studies post COVID-19. Pericardial findings on CMR possibly associated with pericarditis include effusion, thickening, inspiratory septum shift, edema on T2 weighted imaging, and LGE [45]. Current guideline-based diagnostic criteria for pericarditis treat CMR only as adjunctive evidence and no universally agreed-upon CMR criteria for pericarditis exist [46]. In our experience slight pericardial LGE is very common and by itself not suggestive of pericarditis. Furthermore, the differentiation of pericardium and pericardial fat can be difficult in post-contrast T1-weighted imaging. We use fat–water-separated Late-Enhancement-Imaging (mDIXON) to address this problem, although fat–water-swaps do occasionally occur. Visualization of the pericardium as a dark structure in the fat images facilitates differentiation. In our institution, we consider marked pericardial enhancement, or moderate pericardial enhancement in combination with either pericardial thickening or edema on T2 weighted imaging, as suggestive of acute pericarditis. While CMR studies in non-COVID chest-pain patients implicate an under-diagnosis of pericarditis using clinical criteria, the implications of a CMR-diagnosed pericarditis that lacks the clinical criteria for pericarditis are unknown [47].

For myocarditis post COVID-19-vaccination, the available epidemiological studies did not systematically incorporate CMR into the diagnostic workflow [27, 38]. CMR data is limited to small case series, possibly due to different referral patterns and public perception [28, 29, 48]. Reports of frequent myocarditis post COVID-19 in mid to late 2020 generated high awareness and lead to frequent referrals for suspected myocarditis even in cases of moderate to low pretest probability [21]. For myocarditis post vaccination, evidence was only beginning to emerge during our study period and referral was mostly confined to cases with strong clinical suspicion, reflected by the high rate of troponin positives in post-vaccination myocarditis case series, including our report (Table 5). Screening of asymptomatic vaccinated persons was not performed after vaccination as has happened after COVID-19 in athletes. In these screening studies after COVID-19, about ¾ of myocarditis cases were asymptomatic and would not have been discovered on clinical suspicion alone. The corresponding number of asymptomatic and oligosymptomatic myocarditis cases after COVID-vaccination is therefore yet unknown, hampering comparisons of incidence rates.

A recent epidemiological study comparing rates of myocarditis after COVID-19 and after vaccination using matched data from the English national health system and the English national immunization database found a 4 to 40 fold higher incidence of myocarditis in the 28 days following COVID-19 compared to 28 days following COVID-19 vaccination [49]. A recent meta-analysis of 11 studies found an incidence of 18.2 perimyocarditis cases per million COVID-19-vaccine doses, lower than that of other vaccines [50]. This would translate to an estimated 82 cases in Berlin in the study period, which seems consistent with our data, given the high hospital density in Berlin.

Cardiac findings other than myocarditis were more common in our post-COVID-19-cohort compared to the post-vaccination-cohort. Cardiac complications after respiratory infections other than COVID are common. The incidence of myocardial infarction rises after pneumonia and vaccination against influenza reduces cardiovascular mortality [51, 52]. The link between influenza and myocarditis is less clear and CMR-studies are missing [53]. Myocarditis after influenza vaccination is exceedingly rare [54].

### Evolving diagnostic criteria for myocarditis

The diagnosis of myocarditis can be challenging due to its heterogeneous presentation and evolving diagnostic criteria [55]. A definite diagnosis requires histological confirmation via endomyocardial biopsy or post-mortem examination [55]. The 2013 position statement by the European society of cardiology (ESC) suggests a diagnosis of clinically suspected myocarditis in the presence of clinical symptoms and at least one of four clinical criteria, one of which is evidence of LGE and/or edema on CMR [56]. In asymptomatic cases, at least two clinical criteria are required. While CMR has good diagnostic accuracy in “Infarct-like” myocarditis, presenting with fever, chest pain, cardiac enzyme elevations, and ST-segment elevations on ECG, its accuracy is markedly reduced in cardiomyopathic and arrhythmic presentations [57]. The CMR criteria for myocarditis are evolving as well, with the removal of “early gadolinium enhancement” and the introduction of mapping techniques in the updated LCC 2018. These mapping-techniques are also still evolving, with site-dependent normal values and an incomplete understanding of confounders such as sex, age, comorbidities and artifacts [58, 59]. CMR-based strain measurements such as feature tracking and SENC show promise as additional diagnostic and prognostic parameters in myocarditis but have yet to be incorporated into official recommendations. (33454266, 32682718) In summary, these factors contribute to a fragmentation of diagnostic approaches: Some groups did not use CMR at all. Some groups required both updated Lake Louise Criteria, others just one. Some groups required matching LGE and edema and some excluded LGE on the RV-insertion-site. This, in combination with referral bias, mostly solves the conundrum of widely varying myocarditis rates between centers. To demonstrate this point and facilitate comparison of our results with other studies, we reassessed our myocarditis cases based on different diagnostic criteria (Table 7).

**Table 7:** Number of diagnoses based on different CMR Criteria for acute myocarditis

Criterion	Post-COVID-19 (N = 104)	Post-vaccination (N = 27)
Nonischemic LGE + edema	7 (6.7%)	9 (33.3%)
Nonischemic LGE + matching edema	5 (4.8%)	9 (33.3%)
Nonischemic LGE + matching edema, excluding RV-insertion-site	2 (1.9%)	9 (33.3%)

*CMR* cardiac magnetic resonance, *LGE* late gadolinium enhancement, *RV* right ventricle, – no abnormality detected, *AFlu* atrial flutter, *AZ* AstraZeneca, *ECV* extracellular volume, *LBBB* left bundle branch block, *LGE* late gadolinum enhancement, *LVEF* left ventricular ejection fraction, *N/A* not available, *PE* pericardial effusion, *Pt* patient, *RV* right ventricle, *RVEF* right ventricular ejection fraction, *ST* sinus tachycardia, *STE* ST-segment-elevation, *WMA* wall motion abnormalities

## Limitations

Due to infectiosity the post-COVID-19-patients were not examined during the acute stage of the disease but at a mean of 112 ± 76 days after the first positive PCR test. Therefore, a myocarditis at the beginning of the COVID-19 disease might no longer show edema at the time of CMR. Myocardial damage as assessed by LGE on the other hand is usually permanent and would still be visible at the time of CMR as a sign of subsided myocarditis. We found only three cases of possible subsided myocarditis compared to nine cases with edema still present in the post-COVID-19 cohort, which indicates that most cases of myocarditis were still in their acute or subacute phase. In the post-vaccination-cohort, time from vaccination to CMR was rather long as well with 44 ± 35 days. We attribute this to the low awareness of myocarditis as a possible complication of vaccination during the examination period and the inclusion of patients with unremarkable basic cardiac workup but intractable symptoms, who underwent a period of watchful waiting.

Examinations were performed on a 3T and a 1.5T scanner. Because T1 and T2 relaxation times differ between field strengths, comparisons of those can only be drawn within the same field strength.

Our patients represent an unselected cohort of clinical all-comers, therefore confounders in patient selection might be present. As our clinic sees only a part of myocarditis-patients in Berlin, our numbers do not and cannot reflect the true prevalence of myocarditis both post COVID-19 and post vaccination. Our findings suggest a different referral pattern between both groups at the time, with a lower threshold for CMR in post-COVID-19-patients compared to post-vaccination-patients. Patients after vaccination presented with higher cardiac enzymes and had more evident clinical symptoms at the time of the examination.

## Conclusion

Despite the manifold higher number of vaccinations than COVID-19-infections, more patients after COVID-19 than after vaccination presented to our clinic with suspected cardiac involvement. Of those, CMR offered a diagnosis with therapeutic relevance in 32.7% of patients after COVID-19 and 81.5% of patients after vaccination. Applying stricter CMR criteria for myocarditis reduced cases post-COVID-19 but not post vaccination, reflecting more atypical myocarditis presentations post-COVID-19. Further epidemiological studies are needed to refine diagnostic criteria and determine the true prevalence and type of heart damage related to both COVID-19-disease and COVID-19-vaccines.

## Data Availability

The datasets used and/or analysed during the current study are available from the corresponding author on reasonable request.
